# Development and internal validation of a simple clinical score for the estimation of the probability of deep vein thrombosis in outpatient emergency department patients

**DOI:** 10.1016/j.rpth.2024.102608

**Published:** 2024-10-29

**Authors:** Thor-David Halstensen, Camilla Hardeland, Waleed Ghanima, Vigdis Abrahamsen Grøndahl, Aliaksandr Hubin, Mazdak Tavoly

**Affiliations:** 1 Faculty of Health, Welfare and Organisation, Østfold University College, Fredrikstad, Norway; 2 Institute of Clinical Medicine, University of Oslo, Oslo, Norway; 3 Internal Medicine Clinic, Østfold Hospital Trust, Sarpsborg, Norway; 4 Department of Hematology, Oslo University Hospital and Institute of Clinical Medicine, University of Oslo, Oslo, Norway; 5 Østfold University College, Fredrikstad, Norway; 6 Department of Mathematics, University of Oslo, Oslo, Norway; 7 Department of Medicine, Sahlgrenska University Hospital, Gothenburg, Sweden

**Keywords:** D-dimer, deep vein thrombosis, diagnosis, clinical decision rules, probability

## Abstract

**Background:**

Wells score comprises subjective elements, making physicians reluctant to use Wells score or cause them to use it incorrectly.

**Objectives:**

To develop and internally validate a prediction score that is objective and simple for evaluating suspected deep vein thrombosis (DVT), with a safety comparable with that of Wells score.

**Methods:**

We performed a post hoc analysis using data from the Ri-Schedule study (NCT02486445) involving suspected DVT patients at Østfold Hospital’s Emergency Department, Norway (2015-2018). Candidate variables were identified through bootstrapping technique, with a confirmed DVT diagnosis as the outcome variable. Sensitivity, specificity, negative predictive value (NPV), and positive predictive values (PPV) were estimated and compared with the 2-tier Wells score.

**Results:**

Among 1312 patients (median age, 64 years [IQR, 52-73]; 55% women), 19.9% were diagnosed with DVT. Exploration of 30 variables identified tenderness along deep veins and previous venous thromboembolism as significant predictors (selection frequency >60% in 1000 bootstrapping samples). The derived score categorized 450 patients with 0 items as unlikely to have DVT, of whom 8.0% were diagnosed with DVT, compared with 8.2% in DVT unlikely category according to Wells score. Compared with Wells score, the derived score demonstrated sensitivity of 86.2 (95% CI, 81.4-90.2) vs 80.1 (95% CI, 74.7-84.8), specificity of 39.4 (95% CI, 36.4-42.4) vs 55.3 (95% CI, 52.2-58.3), NPV of 92.0 (95% CI, 89.4-94.0) vs 91.8 (95% CI, 89.7-93.5), and PPV of 26.1 (95% CI, 24.8-27.5) vs 30.8 (95% CI, 28.9-32.8). When incorporating D-dimer cutoff of <0.5 µg/mL, the derived score had sensitivity of 99.6 (95% CI, 97.9-99.9), specificity of 16.1 (95% CI, 13.1-18.4), NPV of 99.4 (95% CI, 96.0-99.9), and PPV of 22.8 (95% CI, 22.3-23.3).

**Conclusion:**

The derived DVT score, with 2 objective variables, had a comparable safety with that of the Wells score. However, an external validation is mandated prior to clinical use.

## Essentials

• Physicians may hesitate to use the Wells score due to one of its subjective categories.
• Post hoc analysis of data from a prior management study at Østfold Hospital Trust, Norway, was performed.
• An objective score with only 2 categories was derived and internally validated.
• The new score was equally as safe as the Wells score.

## 1 Introduction

The diagnosis of deep vein thrombosis (DVT) is a multistep process that starts with clinical assessment, either by gestalt or a prediction rule, followed by D-dimer testing. Whole-leg compression ultrasound (CUS) is the final step in confirming the presence or absence of DVT [1].

Although several DVT prediction scores have been developed and validated, the Wells score is still the most widely used prediction score worldwide. Both the original and simplified versions of the Wells score have been thoroughly validated and are often regarded as reference standards to which other clinical prediction scores are compared. However, most available scores include a subjective category (clinical gestalt) that may be difficult to standardize [[2], [3], [4], [5], [6]]. Moreover, a subjective category, eg, “alternative diagnosis more likely/DVT most likely diagnosis,” is often dependent on the assessor’s risk appetite and experience [2,7,8]. Consequently, healthcare providers may be reluctant to use the score in clinical practice or may use it incorrectly.

Recently, it has been debated that the derivation of available scores has not followed modern recommended methodological principles, eg, bootstrapping techniques [[9], [10], [11]]. Furthermore, it has been advocated that D-dimer should be included as a candidate variable when exploring possible predictors for DVT [11]. Accordingly, the objective of the present study was to develop and internally validate a simple and objective clinical prediction score for the diagnosis of DVT with no more than 5 variables, with safety comparable with that of the Wells score, using current recommended methodological principles.

## 2 Methods

### 2.1 Study design and participants

The present study was a post hoc analysis based on data from the rivaroxaban for scheduled work-up of DVT study (Ri-Schedule; NCT02486445) [12]. Ri-Schedule was a prospectively designed outcome study including consecutive patients with suspected DVT referred to the Emergency Department at Østfold Hospital Trust, Norway, between 2015 and 2018. The Ri-Schedule study’s primary objective was to determine the safety of rivaroxaban in the prediagnostic phase of DVT. All participating patients in the Ri-Schedule study were followed up 90 days after inclusion. However, based on available literature and clinical plausibility, the Ri-Schedule study registered patient-related, clinical, and biochemical variables deemed as potential predictors of DVT to be explored for the refinement of DVT management.

All patients included in the Ri-Schedule study were screened for eligibility in this study. The inclusion criteria for the present study were patients aged ≥18 years and referred for a suspected first or recurrent DVT in the lower extremities. Patients who did not provide consent, who were on anticoagulants for indications other than empirical anticoagulation for suspected DVT at the time of inclusion, and who were prescribed anticoagulants for indications other than venous thromboembolism (VTE) in the interval from inclusion until the end of the 3-month follow-up were excluded. In addition, patients who did not have available D-dimer results at baseline were excluded.

The 2- and 3-tier Wells scores were assessed in all patients. Blood test results, including D-dimer (STA-Liatest D-Di Plus; Stago Diagnostics), were analyzed in all patients. A cutoff of ≥0.5 µg/mL fibrinogen equivalent units was considered a positive test result. Patients with positive D-dimer were referred for whole-leg CUS. The deep and saphenous veins were scanned with a linear probe (5-10 MHz). For first DVT, recurrent contralateral DVT, recurrent ipsilateral DVT with documented resorption of thrombus, or recurrent DVT without available images for comparison, the diagnostic criterion was incompressibility of the vein or a grayscale visualization of the thrombus [8]. Recurrent ipsilateral DVT was defined as noncompressibility of, or visualization of, the thrombus in a venous segment not involved from reference CUS.

Patients were discharged from the emergency department without treatment when DVT was considered absent, ie, D-dimer <0.5 µg/mL fibrinogen equivalent units or negative CUS findings. Patients in whom DVT was ruled out by D-dimer or negative CUS findings were contacted by phone on day 90 to assess potential VTE occurrence.

### 2.2 Study variables

Available data in the Ri-Schedule study regarding the following variables were collected: age, sex (male/female), symptom duration, previous VTE (defined as previous DVT or pulmonary embolism), VTE in first-degree relatives, 2- and 3-tier (original 9 items) Wells score, history of thrombophilia including activated protein C resistance (heterozygous and homozygous), antithrombin deficiency, protein C deficiency, protein S deficiency, presence of antiphospholipid antibodies or antiphospholipid syndrome, prothrombin gene mutations, orthopedic surgery in the prior 12 weeks, other surgery in the prior 12 weeks, immobilization due to trauma (defined as trauma requiring hospital admission or plaster immobilization within the prior 12 weeks), immobilization due to neurologic disease (defined as presence of neurologic disease with paresis of the lower extremities), long-haul (>4 hours) flight, long-haul (>4 hours) travel by car or train, suspected infection (fever, chills, sweating, reduced general condition, or clinical suspicion of erysipelas/cellulitis), pain, red discoloration (of the lower extremity), hormone replacement therapy, pregnancy, puerperium, oral contraceptives, myeloproliferative neoplasms, body mass index (BMI; in kilogram per meter square and dichotomized at BMI <30 or BMI ≥30), and D-dimer. All these variables were assessed as possible candidate variables, ie, predictors of DVT, in this study. However, since our aim was to develop an objective score, when exploring possible predictors of DVT, the category of “alternative diagnosis” in the Wells score was omitted because of the subjectivity of the criteria.

### 2.3 Statistical analysis

All analyses were performed using the statistical software programs STATA 17 (Stata Statistical Software: Release 17 StataCorp LLC), with the external package validation, and R Statistical Software (v4.2.1; R Core Team 2021). *P* values were 2-tailed, and statistical significance was defined as *P* < .05.

Categorical variables are presented as frequencies with percentages and continuous variables as medians with corresponding IQRs.

### 2.4 Derivation of the score

In all analyses, a confirmed DVT diagnosis by positive CUS findings was used as the outcome variable. The maximum number of included variables was estimated according to the rule of 1 variable per 5 to 10 events [13].

The dataset was randomly split into an inference set of 1000 patients for development and a hold-out set of 312 patients for internal validation of the score. To select DVT predictors, 1000 bootstrapped samples were derived from the inference set [9]. Within the 1000 bootstrapped samples, a bootstrap training and testing set was created. Every bootstrap sample randomly selected observations with replacements from the inference set of 1000 patients. If an observation was included in the bootstrapped training set, it was excluded from the bootstrapped testing set, thereby ensuring independence between the 2 sets [14]. Within each bootstrap sample, a stepwise forward-backward logistic regression model with the Bayesian information criterion as the variable selection criterion was deployed. Bootstrapped distributions of the effect sizes were obtained across 1000 selected models. The variables that had a non-0 median effect size (also corresponding to a bootstrapped probability of selection above 0.6) were selected for the final model [15]. Variables present in a minimum of 60%, ie, a frequency of ≥0.6 of the bootstrapped samples, were selected for the final model [15,16]. Eliminating candidate variables before statistical analysis may set the regression coefficient of that variable to 0, thus moving away from maximum likelihood and possibly affecting another variable either positively or negatively. Consequently, this approach may lead to a suboptimal model [15]. In this context, we included D-dimer as a candidate variable in the analysis of exploring possible predictors to compose the score, ie, D-dimer was forced in all models exploring candidate variables as predictors of DVT.

### 2.5 Validation of the score

Since our intention was to develop a dichotomized score in which DVT could be classified as unlikely or likely, we arbitrarily defined DVT a priori to be classified as unlikely according to the score if the prevalence of confirmed DVT was less than 10%. We validated the performance of the score by assessing and comparing the sensitivity, specificity, negative predictive value (NPV) and positive predictive value (PPV), and failure rate with the Wells score in all subjects (*n* = 1312).

Sensitivity was defined as the proportion of patients with confirmed DVT by positive CUS findings who were classified as having DVT likely by the derived score. Specificity was defined as the proportion of patients without DVT, ie, negative D-dimer or negative CUS findings, classified as having DVT unlikely by the derived score. NPV was defined as the proportion of patients classified as having DVT unlikely with the derived score in whom DVT was ruled out by D-dimer or negative CUS findings; PPV was defined as the proportion of patients classified as having DVT likely by the derived score who had DVT confirmed by positive CUS findings. The failure rate was defined as the proportion of patients in whom DVT was ruled out according to the derived score and D-dimer at baseline but who developed a DVT, died, or were lost to follow-up during the 90-day follow-up period of all patients who had DVT predicted as unlikely and a negative D-dimer.

The discriminatory value of the developed score was evaluated in both the inference set (*n* = 1000) and the hold-out set (*n* = 312) using the area under the receiver operating characteristic curve, which was calculated for every bootstrapped testing set. The agreement between the estimated and observed number of events according to the derived score was evaluated with calibration slopes in the inference and hold-out sets.

All the abovementioned statistical measures of the derived score were compared with those of the 2-tier Wells score. For a better understanding of how the derived score performed against the Wells score, we compared the 2 scores both with and without the addition of D-dimer.

Missing data were addressed with case-wise deletion, which excluded all participants with missing values from the analysis if the missing data were present in more than 5% of the study cohort.

### 2.6 Ethical considerations

The Regional Committee for Medical and Health Research Ethics (reference number 2014/377) and the Data Protection Officer at Østfold Hospital Trust approved this study.

## 3 Results

### 3.1 Baseline results

From February 2015 to November 2018, 1653 patients were included in the Ri-Schedule study. Of these, 249 patients were excluded because of ongoing anticoagulant treatment, and an additional 92 patients were excluded because of missing data: BMI (*n* = 52), Wells score (missing in one or several items; *n* = 14), red discoloration (*n* = 10), suspected infection (*n* = 8), D-dimer (*n* = 7), and VTE in first-degree relative (*n* = 1). Thus, the final study population consisted of 1312 patients.

Median age was 64 years (IQR, 52-73) and 55% were women. Two hundred sixty-one (19.9%) patients were diagnosed with DVT. Patient characteristics and risk factors are summarized in Table 1.

**Table 1:** Patient characteristics and risk factors for DVT.

Variable	All *n* = 1312	Confirmed DVT *n* = 261	Inference set *n* = 1000	Hold-out set *n* = 312
Age (y), median (IQR)	64 (52-73)	64 (52-73)	64 (52-72)	64 (51-73)
Female sex, *n* (%)	723 (55)	107 (41)	551 (55)	172 (55)
Symptom duration (d), median (IQR)	7 (3-14)	5 (3-7)	7 (3-14)	7 (3-14)
Previous VTE, *n* (%)	187 (14)	69 (26)	137 (14)	50 (16)
VTE in first-degree relatives, *n* (%)	250 (19)	54 (21)	186 (19)	64 (21)
Active cancer,^a^*n* (%)	61 (5)	21 (8)	47 (5)	14 (4)
Paralysis, paresis, or recent plaster immobilization of lower extremity, *n* (%)	53 (4)	13 (5)	40 (4)	13 (4)
Bedridden recently, *n* (%)	86 (7)	27 (10)	59 (6)	27 (9)
Tenderness along the deep venous system, *n* (%)	808 (62)	209 (80)	607 (61)	201 (64)
Entire leg swollen, *n* (%)	269 (21)	71 (27)	203 (20)	66 (21)
Calf swelling >3 cm, *n* (%)	342 (26)	113 (43)	264 (26)	78 (25)
Pitting edema confined to the symptomatic leg, *n* (%)	577 (44)	161 (62)	440 (44)	137 (44)
Collateral superficial veins present, *n* (%)	154 (12)	41 (16)	118 (12)	36 (12)
Known thrombophilia,^b^*n* (%)	36 (3)	14 (5)	24 (2)	12 (4)
Recent surgery, *n* (%)	104 (8)	31 (12)	81 (8)	23 (7)
Immobilization due to trauma, *n* (%)	64 (5)	17 (7)	45 (5)	19 (6)
Immobilization neurologic disease, *n* (%)	28 (2)	7 (3)	20 (2)	8 (3)
Travel >4 h, *n* (%)	373 (28)	79 (30)	274 (27)	99 (32)
Suspected infection,^c^*n* (%)	73 (6)	6 (2)	58 (6)	15 (5)
Pain, *n* (%)	1117 (85)	228 (87)	844 (84)	273 (88)
Red discoloration, *n* (%)	393 (30)	93 (36)	298 (30)	95 (30)
Hormone replacement therapy, *n* (%)	118 (9)	15 (6)	93 (9)	25 (8)
Pregnancy or puerperium, *n* (%)	19 (1)	1 (0)	17 (2)	2 (1)
Hormonal contraceptives, *n* (%)	35 (3)	8 (3)	23 (2)	12 (4)
Myeloproliferative neoplasms, *n* (%)	6 (0.5)	0 (0)	4 (0.4)	2 (0.6)
Body mass index >30 kg/m^2^, *n* (%)	479 (37)	91 (35)	366 (37)	113 (36)
D-dimer (µg/mL), median (IQR)	0.8 (0.4-1.7)	3.3 (0.8-7)	0.8 (0.4-1.7)	0.7 (0.4-1.6)
DVT likely,^d^*n* (%)	633 (48)	209 (80)	480 (48)	153 (49)
DVT unlikely,^d^*n* (%)	679 (52)	52 (20)	520 (52)	159 (51)
Low probability for DVT,^e^*n* (%)	363 (28)	25 (10)	284 (28)	79 (25)
Moderate probability for DVT,^e^*n* (%)	625 (48)	114 (44)	466 (47)	159 (51)
High probability for DVT,^e^*n* (%)	324 (25)	112 (47)	249 (25)	75 (24)
DVT, *n* (%)	261 (20)	NA	198 (20)	63 (20)
DVT proximal, *n* (%)	174 (67)	NA	129 (65)	45 (71)
DVT distal, *n* (%)	87 (33)	NA	69 (35)	18 (29)

DVT, deep vein thrombosis; NA, not applicable; VTE, venous thromboembolism.

a Active cancer: cancer diagnosed in the previous 6 months.

b Thrombophilia: activated protein C resistance (heterozygote and homozygote), antithrombin deficiency, protein C deficiency, protein S deficiency, presence of antiphospholipid antibodies or antiphospholipid syndrome, and reduced prothrombin levels.

c Suspected infection: fever, chills, sweating, reduced general condition, or clinical suspicion of erysipelas/cellulitis.

d According to the modified 2-tier Wells score.

e According to the original 3-tier Wells score.

### 3.2 Variable selection and derivation of the derived score

Confirmed DVT in 261 cases allowed around 30 variables to be explored as possible predictors for DVT. Table 2 provides the bootstrapping effects of the selected variables. Localized tenderness along the deep venous system (selection frequency, 0.89; median effect size, 0.96) and history of venous thromboembolism (selection frequency, 0.84; median effect size, 1.04) were the 2 variables that reached the predefined selection frequency of 0.6 (selected in at least 60% of the bootstrapped samples) and were thus included in the derived score.

**Table 2:** Bootstrapping effect of the included variables.

Variable^a^	Frequency	Median effect size	95% CI
Intercept	1.0000	−4.1015	−5.1335 to 2.5036
D-dimer	1.0000	0.8162	0.6040 to 1.0928
Tenderness along the deep venous system	0.8880	0.9559	0.0000 to 1.5189
Previous VTE	0.8430	1.0398	0.0000 to 1.5719
Sex	0.5220	0.5879	0.0000 to 1.0589
Immobilization due to trauma	0.3060	0.0000	0.0000 to 1.7978

VTE, venous thromboembolism.

a For brevity, only variables with frequencies >0.3 are presented.

Among the 1312 participants enrolled, 450 patients (34%) had neither tenderness nor previous VTE. Conversely, 729 patients (56%) demonstrated either tenderness or a prior VTE, while 133 patients (10%) had both tenderness and previous VTE. DVT was confirmed in 36 patients (8%) with 0 items present, in 172 patients (24%) with 1 item present, and in 53 patients (40%) with 2 items present. According to the prevalence of confirmed DVT in each of the groups, patients who had 0 items present were classified as having DVT unlikely (prevalence of DVT less than 10%), whereas those with at least 1 item present were classified as having DVT likely (Table 3). Thus, DVT was considered unlikely in (450/1312) 34% and likely in 66%.

**Table 3:** The proportion of patients and prevalence of DVT in each score category of the derived score, modified Wells score, and original Wells score.

Risk score	All patients *N* = 1312	Number and proportion with confirmed DVT *n* = 261
Derived DVT score		
0 items, n (%)	450 (34)	36 (8.0)
1 item, n (%)	729 (56)	172 (23.6)
2 items, n (%)	133 (10)	53 (39.8)
DVT unlikely, n (%)	450 (34)	36 (8.0)
DVT likely, n (%)	862 (66)	225 (26.1)
Modified Wells score^a^		
DVT unlikely,^b^ n (%)	633 (48)	52 (8.2)
DVT likely,^b^ n (%)	679 (52)	209 (30.8)
Original Wells score^b^		
Low probability of DVT, n (%)	363 (28)	25 (6.8)
Moderate probability of DVT, n (%)	625 (48)	114 (18.2)
High probability of DVT, n (%)	324 (25)	122 (37.9)

DVT, deep vein thrombosis.

a According to the modified 2-tier Wells score.

b According to the original 9-item 3-tier Wells score.

### 3.3 Comparison with the Wells score

The modified Wells score categorized DVT as unlikely in 633 patients (48%) and as likely in 679 patients (52%). DVT was confirmed in 8.2% and 30.8% of the patients in each group, respectively (Table 3).

### 3.4 Prevalence of DVT by items present in the derived DVT score and D-dimer categories

**Figure 1:**
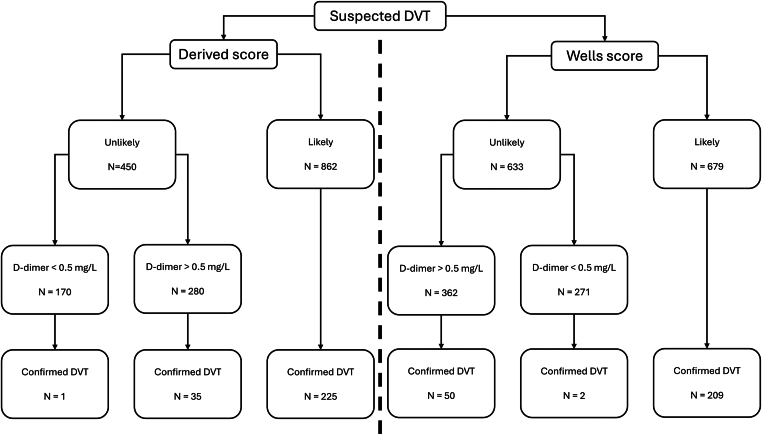
Study flow and outcomes. DVT, deep vein thrombosis.

Similar to the Wells score, when incorporating D-dimer at a threshold of 0.5, the derived score missed 1 of 170 patients with confirmed DVT when DVT was deemed unlikely and D-dimer was <0.5 µg/mL (Table 4, Figure 1).

**Table 4:** Prevalence of confirmed DVT according to the probability as assessed by the derived and Wells score according to D-dimer results (*n* = 1312).

Derived score	D-dimer < 0.5 (µg/mL)		D-dimer ≥ 0.5 (µg/mL)	
	Derived score	Wells score^a^	Derived score	Wells score^a^
DVT unlikely	1/170	2/271	35/280	50/362
DVT likely	2/219	1/118	223/643	208/561

DVT, deep vein thrombosis.

a Two-tier Wells score.

### 3.5 Performance of the derived score compared with the Wells score

Table 5 displays the performance of the derived score compared with the 2-tier Wells score. The crude performance, ie, without D-dimer, was similar between the 2 scores. Adding D-dimer increased the NPV of the derived score to 99.4 (95% CI, 96.0-99.9). One patient out of the 170 who was classified as having DVT unlikely and negative D-dimer according to the derived score was diagnosed with a proximal DVT during the 90-day follow-up (Figure 1). In addition, 7 patients died during the 90-day follow-up period. However, autopsy was not performed in all cases. Assuming that the cause of death was related to missed VTE diagnosis in all cases that could increase the failure rate, but all the patients who died had a positive D-dimer of ≥0.5 µg/mL and 5 of the patients who died would be classified as unlikely to have DVT according to the derived score. Two patients were lost to follow-up. They both had negative D-dimer and were subjected to CUS at baseline. In summary, among the patients with negative D-dimer and deemed unlikely to have DVT according to the new score, 1 was missed and 2 were lost to follow-up, resulting in a failure rate of 3 of 170, 1.8% (0.0-3.7). In comparison, the failure rate for the Wells score was up to 4 of 271, 1.5 (0.0-2.9).

**Table 5:** Performance indices of the derived score compared with the modified Wells score in all patients (*n* = 1312).

Performance	Without D-dimer		With D-dimer < 0.5 (µg/mL)	
	Derived score Value (95% CI)	Wells score^a^ Value (95% CI)	Derived score Value (95% CI)	Wells score^a^ Value (95% CI)
Sensitivity	86.2 (81.4-90.2)	80.1 (74.7-84.8)	99.6 (97.9-99.9)	99.2 (97.3-99.9)
Specificity	39.4 (36.4-42.4)	55.3 (52.2-58.3)	16.1 (13.1-18.4)	24.4 (21.9-27.1)
NPV	92.0 (89.4-94.0)	91.8 (89.7-93.5)	99.4 (96.0-99.9)	99.2 (96.9-99.8)
PPV	26.1 (24.8-27.5)	30.8 (28.9-32.8)	22.8 (22.3-23.3)	24.6 (23.9-25.3)

NPV, negative predictive value; PPV, positive predictive value.

^a^Two-tier Wells score.

### 3.6 Internal validation

**Figure 2:**
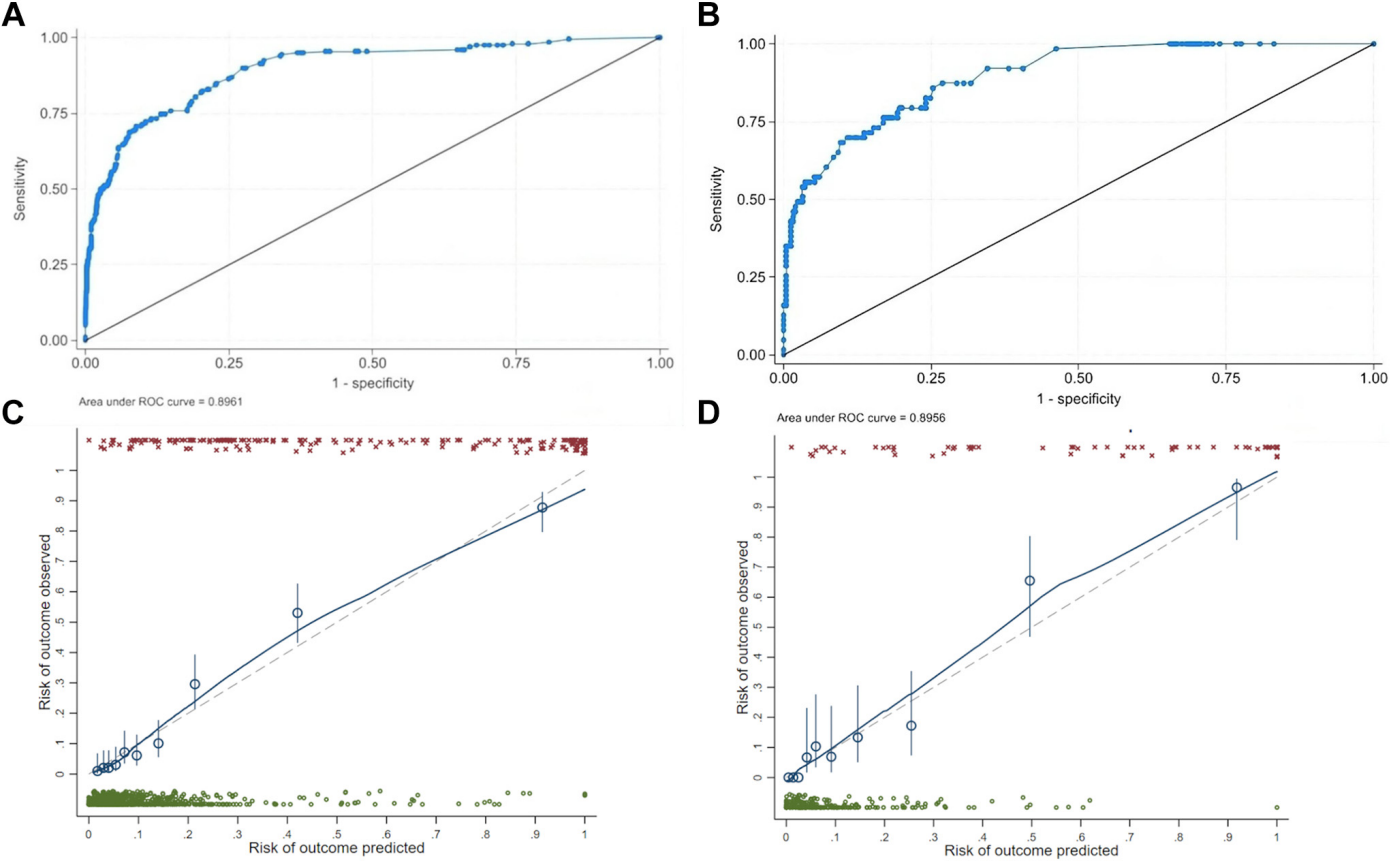
Area under the ROC curves and calibration estimates of the derived score. ROC curves and calibration curve of our new prediction model. (A) ROC curve and corresponding area under curve for the prediction model of deep vein thrombosis diagnosis in the inference set. (B) ROC curve and corresponding area under curve for the prediction model of deep vein thrombosis diagnosis in the hold-out set. (C) The calibration curve of the inference set. (D) The calibration curve of the hold-out set. ROC, receiver operating characteristic.

Figure 2 displays the discriminatory performance of the derived score in terms of area under the receiver operating characteristic curves and calibration slopes estimated in the inference set (Figure 2A, C) and the hold-out set (Figure 2B, D). The area under the curve was 0.89 in both the inference and hold-out sets, whereas the calibration slope was 0.98 (95% CI, 0.75-1.19) in the inference set and 0.92 (95% CI, 0.56-1.35) in the hold-out set, indicating adequate internal validity (Figure 2).

## 4 Discussion

This study aimed to develop and internally validate a simple and objective clinical prediction score for the diagnosis of DVT. Among 30 candidate variables, we found tenderness along the deep venous system and previous VTE to be significant predictors of DVT. The derived new score was found to have a safety comparable with that of the Wells score and adequate internal validity.

Most previous studies developing clinical prediction scores for VTE have used a traditional statistical methodology, ie, selecting possible predictors of the outcome variable by univariable and multivariable regression analyses. In this study, a bootstrapping technique was used to select predictors of DVT, which has been suggested as a more robust variable selection technique [9,17]. We found tenderness along the course of the deep veins and previous history of VTE as the 2 most frequently occurring variables in the bootstrapped samples. Because the variable selection method is different from that used in previous studies, comparison may be difficult. However, historical data support both tenderness along the course of the deep veins and history of VTE as strong predictors of DVT [[18], [19], [20], [21]]. In contrast, a recent study based on individual patient data from several other studies that also used a bootstrapping technique did not find tenderness and previous VTE as predictors of DVT [22]. Notably, 2 of the studies from which data were derived included the 9-item Wells score, which excludes the item “previous history of VTE.” In addition, and in contrast to our study, the subjective category of “DVT as the most likely diagnosis/another diagnosis more likely” was included when selecting predictors. To our surprise, calf or entire leg swelling were not found to be potential predictors in the bootstrapped observations. One explanation may be the exclusion of the implicit category of “DVT as the most likely diagnosis/another diagnosis more likely,” which may have affected the effect size of other candidate variables. However, one of the main objectives of this study was to develop a score without a subjective item.

In this study, D-dimer was included in the variable selection process. This approach contrasts with previously used statistical methods. It has been debated whether D-dimer should be included in the candidate variable selection process [11]. From a methodological point of view, we believe that when analyzing possible candidate variables, D-dimer should be included as it has a huge impact on the outcome event of VTE. However, to the best of our knowledge, studies supporting this approach are unavailable.

According to the derived score, 8% of patients classified as having DVT unlikely had a DVT confirmed. A similar proportion was observed when classifying according to the Wells score, which resulted in comparable sensitivity and NPV between the 2 scores. The derived score showed a comparable prevalence of confirmed DVT with the Wells score (1/170 vs 2/271) in cases where DVT was deemed unlikely and D-dimer was negative. This improved failure rate came at the cost of more patients being referred for CUS (increased false positives compared with the Wells score). However, the primary aim of this study was to develop a simple and objective score with adequate safety. Moreover, we believe that improving specificity is more likely achieved with an increased cutoff of the D-dimer test rather than with the prediction score itself.

The strength of the present study is its design based on prospectively collected data. Furthermore, the current recommended standards for developing prediction rules were used [23]. Also, the derived score was evaluated regarding the calibration performance, which is recommended to be performed when developing prediction scores. In this context, to our knowledge, only one previous study has provided calibration measures related to VTE prediction scores. Finally, reporting a DVT prevalence of 19.9% indicates the study sample was representative of a true DVT population [22,24], thereby reducing the risk of overestimating the performance of a clinical probability score.

This study has several limitations that merit mentioning. Although we adhered to the recommendations from the literature regarding the total number of candidate variables included, evaluating 30 variables might have been overly optimistic. However, including 10 candidate variables per event (as recommended in the literature), rather than the 9 per event utilized in this study, would probably have had only a minor impact on the results. The dataset was split into an inference and a hold-out set. This approach has been questioned by some as being insufficient when selecting predictor variables since not all available data are used [23]. Bootstrapping technique was used for candidate variable selection. Therefore, although we have conducted the analysis according to recent methodological standards, the results need to be interpreted with caution until an external validation is performed. The increased prevalence of tenderness along the deep veins within this cohort compared with previous studies [20] raises the possibility that the selection of this variable as a significant predictor might have occurred by chance. However, the prevalence of tenderness along the deep veins varies widely in previous studies, which complicates direct comparisons. Furthermore, a recent study on individual patient data (*n* = 3368) reported a prevalence similar to that observed in this study [22].

Unfortunately, data on ethnicity was not recorded. This study analyzed data from a prospective management study conducted at a Norwegian emergency department, including all patients with suspected DVT. Although the patients were primarily Caucasian, as is common in most European countries, individuals with non-Caucasian ethnicity have not been shown to have a higher prevalence of VTE [25,26]. Consequently, we believe the lack of ethnicity data is unlikely to be a significant limitation in this study.

Clinical utility was not evaluated, which perhaps makes it difficult to assess the clinical implication of the derived score. However, in line with recent studies, improved clinical utility is perhaps mostly achieved by incorporating D-dimer into the clinical score or increasing the D-dimer threshold and not by developing a clinical score [21,22]. To this end, our primary aim was to first develop a simple and objective score with safety comparable with that of the Wells score. Furthermore, as we could not distinguish between acute and chronic DVT in cases with DVT recurrence in the ipsilateral leg, some cases might have been incorrectly regarded as acute DVT when in fact they were chronic. Finally, while the derived score demonstrated safety comparable with that of the Wells score, it is essential to recognize that its performance was evaluated within the same cohort from which it was derived. Hence, the true performance of the derived score, as well as its comparison to the Wells score, remains to be established in an external validation study.

## 5 Conclusion

We developed and internally validated a new score with safety comparable with that of the Wells score. If externally validated, the derived score has the potential to facilitate the management of DVT patients because of its feasibility and objectivity. However, it is important to note that, in its current form, the new score would increase the number of patients requiring further evaluation by 14% compared with the Wells score.
